# A Simple Method to Measure Renal Function in Swine by the Plasma Clearance of Iohexol

**DOI:** 10.3390/ijms19010232

**Published:** 2018-01-12

**Authors:** Sergio Luis-Lima, Consolación García-Contreras, Marta Vázquez-Gómez, Susana Astiz, Fabiola Carrara, Flavio Gaspari, Natalia Negrín-Mena, Alejandro Jiménez-Sosa, Hugo Jiménez-Hernández, Antonio González-Bulnes, Esteban Porrini

**Affiliations:** 1 Research Unit, University Hospital of the Canary Islands, 38320 La Laguna, Tenerife, Spain; luislimasergio@gmail.com (S.L.-L.); natalianegrinmena@gmail.com (N.N.-M.); ajimenezsosa@gmail.com (A.J.-S.); hjimenezhdez@gmail.com (H.J.-H.); 2 Comparative Physiology Group, SGIT-INIA, 28040 Madrid, Spain; garcia.consolacion@inia.es (C.G.-C.); astiz.susana@gmail.com (S.A.); bulnes@inia.es (A.G.-B.); 3 Faculty of Veterinary, Universidad Complutense de Madrid, 28040 Madrid, Spain; mvgomez@ucm.es; 4 IRCCS-Istituto di Ricerche Farmacologiche Mario Negri, Clinical Research Center for Rare Diseases ‘Aldo & Cele Daccò’, 24020 Ranica (BG), Italy; fabiola.carrara@marionegri.it (F.C.); flavio.gaspari@marionegri.it (F.G.); 5 Instituto Tecnologías Biomédicas (ITB), University of La Laguna, 38320 Tenerife, Spain

**Keywords:** renal function, iohexol plasma clearance, swine model

## Abstract

There is no simple method to measure glomerular filtration rate (GFR) in swine, an established model for studying renal disease. We developed a protocol to measure GFR in conscious swine by using the plasma clearance of iohexol. We used two groups, test and validation, with eight animals each. Ten milliliters of iohexol (6.47 g) was injected into the marginal auricular vein and blood samples (3 mL) were collected from the orbital sinus at different points after injection. GFR was determined using two models: two-compartment (CL2: all samples) and one-compartment (CL1: the last six samples). In the test group, CL1 overestimated CL2 by ~30%: CL2 = 245 ± 93 and CL1 = 308 ± 123 mL/min. This error was corrected by a first-order polynomial quadratic equation to CL1, which was considered the simplified method: SM = −47.909 + (1.176xCL1) − (0.00063968xCL1^2^). The SM showed narrow limits of agreement with CL2, a concordance correlation of 0.97, and a total deviation index of 14.73%. Similar results were obtained for the validation group. This protocol is reliable, reproducible, can be performed in conscious animals, uses a single dose of the marker, and requires a reduced number of samples, and avoids urine collection. Finally, it presents a significant improvement in animal welfare conditions and handling necessities in experimental trials.

## 1. Introduction

Translational studies in animal models are necessary to evaluate the pathogenesis of renal disease. Most of the basic research and preclinical studies in renal pathophysiology have been performed in mice and rats. Rodents need reduced space, are relatively inexpensive to maintain, are easy to manage, have a short life cycle and, in the case of mice, are easily modified by genetic engineering [1]. Thus, during the last decades, rodents have been extensively used as models for human renal disease [2].

However, rodents do not completely recapitulate human renal disease. This has been observed for diabetic nephropathy [3], membranous glomerulonephritis [4], and hemolytic uremic syndrome [5], among other renal diseases. To facilitate translational research, kidney structure and function in animal models should be similar to that in humans. However, rodent kidneys have a single papilla and undivided medulla and cortex. On the other hand, humans and swine share similar features of kidney structure, function, and physiology [6,7,8,9]. Swine kidneys are multipyramidal with a cortex and several different medullary structures; each medullary pyramid forms a separate papilla and their fusion results in the formation of some compound papillae. Also, renal physiology is very similar between swine and man [8], including maximal urine concentration (1080 and 1160 mOsmol/L, respectively), maximal urine-to-plasma osmolality ratio (3.7 and 4.0), glomerular filtration rate (130 and 126–175 mL/min per 70 kg), and total renal blood flow (4 and 3.0–4.4 mL/min per gram). Hence, swine is currently recognized as an amenable model for renal pathology [10,11].

The incidence of chronic kidney disease (CKD) and end-stage renal disease (ESRD) is increasing worldwide [12]. Moreover, age-standardized death rates have increased by 9% for diabetes and 37% for CKD, while those of non-communicable diseases have decreased by 18% [13]. CKD is characterized by a progressive loss in the glomerular filtration rate (GFR). This figure may have several causes, one of which is the lack of a reliable animal model, with an accurate method to evaluate GFR, to study CKD and diabetic nephropathy [14]. This may help in studies on the pathogenesis of CKD and also in the prevention of renal damage by new drugs.

GFR can be measured by the clearance of inulin, radioactively labeled markers such as ^51^Cr–EDTA, ^125^I–iothalamate, and ^99^mTc–DTPA (diethylene-triamine-pentaacetate), or non-radioactive markers such as iohexol [15,16] and iothalamate. Some of these methods are complex and unpractical. Inulin requires continuous infusion of the marker and catheterization of the bladder. The latter requires the use of anesthesia, which could influence GFR and, therefore, alter the results of the experiment. ^51^Cr–EDTA and ^99^mTc–DTPA have the limitation of being radioactive markers. Finally, iothalamate may be affected by the existence of tubular secretion [17,18]. The plasma clearance of iohexol has several advantages in clinical practice and research as recently reviewed [15,16]. This method is simple, reliable, and a safe alternative to evaluate GFR [15,16]. Iohexol is not bound by proteins, is metabolically inert and is freely filtrated by the glomeruli; also, it is neither secreted nor metabolized by tubular cells and has negligible extra-renal clearance [15,16,19]. Moreover, iohexol is safe even in patients with chronic kidney disease [15,16,20].

Very few studies have evaluated the reliability of gold standard methods for evaluating GFR in swine. The use of iohexol has been evaluated in swine [21] and compared with ^51^Cr–EDTA [22]. Frennby in 1997 described plasma and renal clearance of iohexol and ^51^Cr–EDTA in 21 anesthetized swine. In that study, the animals were anesthetized for handling; also, urine collection and multiple blood sampling were needed. A simplified approach to measure GFR was tested in that study, using a correction formula designed for humans, which led to an overestimation of the true GFR. Thus, to the best of our knowledge, there is no simple and reliable method to measure GFR in swine, which limits the usefulness of swine as an animal model for renal disease.

The objective of this study was to develop a method using plasma clearance of iohexol to measure renal function in conscious swine; this would reduce the number of blood samples and also take into consideration animal welfare in accordance with Russell and Burch’s 3Rs model for animal research (refinement, reduction, and replacement) [23].

## 2. Results

### 2.1. Iohexol Plasma Analysis

**Figure 1:**
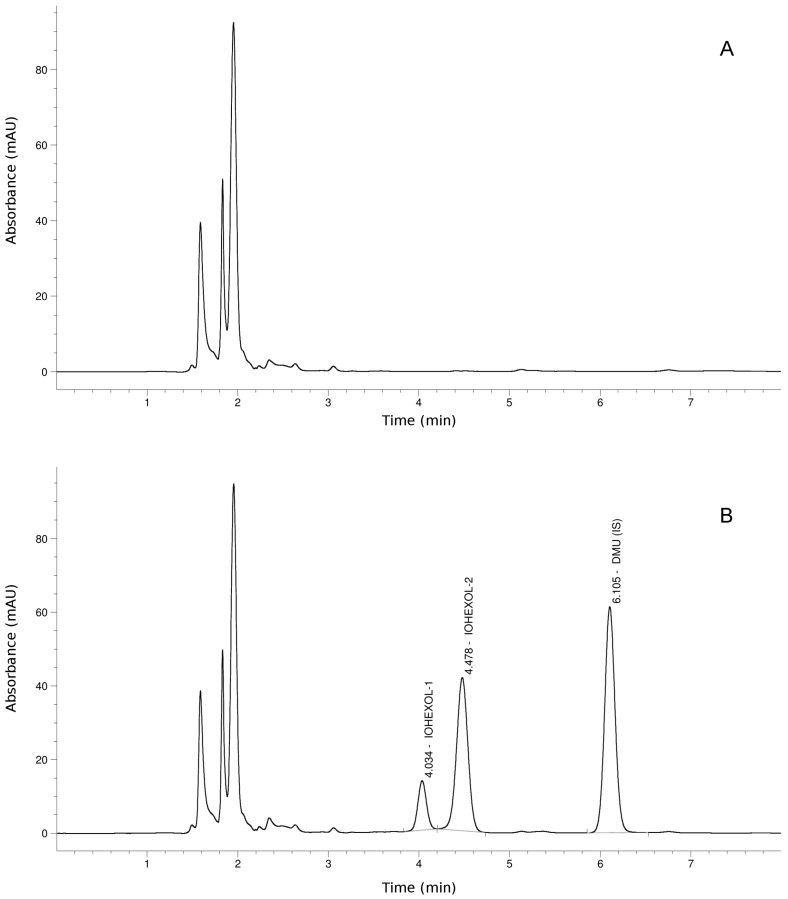
Representative chromatograms of swine plasma before (**A**) and after (**B**) intravenous injection of iohexol (6.47 g). Iohexol isomers and internal standard (IS) 1,3-dimethyluric acid (DMU) were detected at 254 nm.

Figure 1 shows an HPLC–UV chromatogram of an iohexol-free blood sample before injection (Figure 1A) and 120 min after injection (Figure 1B). Iohexol eluted from the chromatographic column as two peaks at 4.03 and 4.47 min, reflecting the isomers present in the pharmacologic preparation. The internal standard 1,3-dimethyluric acid (DMU) eluted at 6.10 min. No interfering peaks were observed in the iohexol-free samples.

### 2.2. Pharmacokinetic Clearance Profiles

**Figure 2:**
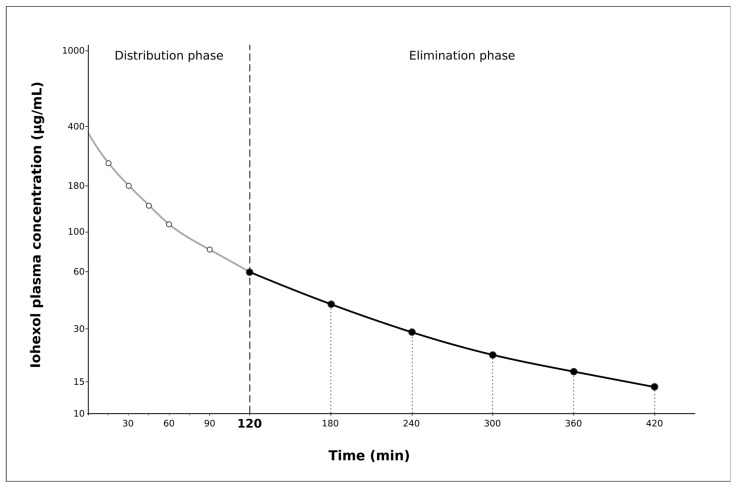
Pharmacokinetic profile of the iohexol plasma clearance considering a two-compartment model (CL2) in representative swine. Sampling time points are indicated by white circles (at 15, 30, 45, 60, and 90 min) for the distribution phase and by black circles (at 120, 180, 240, 300, 360, and 420 min) for the elimination phase. The one-compartment model (CL1) considers only the elimination phase.

Figure 2 shows a two-compartment model for the iohexol plasma clearance. The first part of the curve, from 15 to 120 min, is curvilinear and corresponds to the distribution phase. The second part, from 120 to 420 min, is linear and corresponds to the elimination phase. This is why 120 min was selected as the starting point of the second phase (elimination phase) of the curve. Similar results were observed using 180 min as the starting point.

### 2.3. Test Group

Mean GFR values were 245 ± 93 mL/min (median 229, IQR: 173–331) and 308 ± 128 mL/min (median 278, IQR: 221–411) for CL2 and CL1, respectively (Table 1). In all cases, GFR values determined by CL1 were 20–30% higher than with CL2. The recalculation of CL1 by the Bröchner–Mortensen (BM) equation did not correct this difference. Moreover, this correction led to a systematic underestimation of GFR values that averaged 23% (Table 1).

### 2.4. Correction Formula

The more accurate formula to adjust CL1 to CL2 was the first-order polynomial quadratic equation (y_i_ = a + bx_i_ + cx_i_2; R square: 0.97) (Table S1). Applying this equation, GFR was calculated as follows: the simplified method SM = −47.909 + (1.176xCL1) − (0.00063968xCL1^2^) CL1 is the clearance obtained based on the one-compartment model and SM the recalculated true clearance with the simplified method. GFR in the test group using the SM was 245.4 ± 91.4 mL/min (median: 220, IQR 183–288), which was similar to the value obtained by CL2, which was 245.3 ± 92.8 mL/min. The error was less than 13% for all cases (Table 1). Individual GFR values are shown in Table 1. Finally, the cubic equation was not selected because the difference in GFR between CL2 and CL1 was higher than with the first-order polynomial quadratic formula (Table S1).

### 2.5. Validation Group

The mean GFR values were 229 ± 69 (median: 181, IQR 225–290) mL/min and 277 ± 85 mL/min (median: 267, IQR 224–353) for CL2 and CL1, respectively (Table 1). Applying the Bröchner–Mortensen equation to CL1 led to a 19% underestimation of GFR. On the other hand, GFR assessed by SM was 225 ± 71 mL/min (median: 350, IQR 210–311), which was similar to the value obtained by CL2 (229 ± 69 mL/min), showing an error less than 10% for almost all animals (Table 1). Individual GFR values are shown in Table 1.

### 2.6. Analysis of Agreement

**Figure 3:**
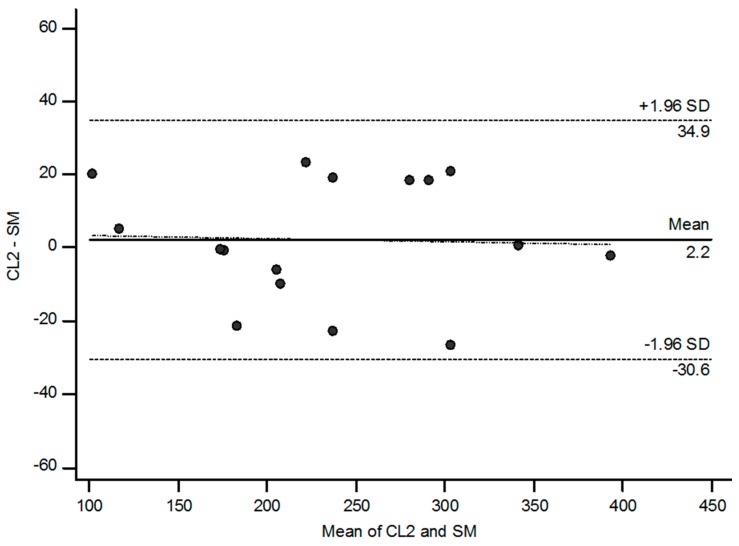
The Bland–Altman plot of the difference between the glomerular filtration rate (GFR) values measured by the reference method (two-compartment clearance: CL2) and simplified method (SM) versus the mean of both. The straight and dashed lines indicate mean difference and 95% limits of agreement, respectively.

The Bland–Altman plot (Figure 3) shows narrow limits of agreement (from −30.6 to 34.9 mL/min) and a mean difference of 2.1 mL/min between values measured with the simplified method (SM) and the reference method (CL2), indicating good agreement.

Compared with the reference method (CL2), the simplified method (SM) had a concordance correlation coefficient (CCC) of 0.97 (0.94, upper confidence interval (CI), reflecting high precision and accuracy. Also, the total deviation index (TDI) was 14.73% (20.62), which means that 90% of the GFR values showed an error ranging from −14.7 to +14.7% when compared with the reference method. Finally, the coverage probability (CP) was 71 (54), which indicates that more than 29% of the GFR values had an error range greater than ±10% of the method in plasma.

### 2.7. Reproducibility Study

The mean absolute percentage error for the replicas in the overall group was 9.3% (Table S2).

### 2.8. Sensitivity Analysis

The results of CL1 were comparable using the starting point of the elimination phase at 120 or 180 min.

### 2.9. Calibration and Quality Control Standards

The differences between the experimental back-calculated concentrations of the calibration standards and the theoretical levels were within ± 5% for all the analyses. The deviations for the low- and high-quality controls were always lower than 7.5%.

## 3. Discussion

The present study offers a new, simple, reliable, and reproducible method using plasma clearance of iohexol to measure glomerular filtration rate (GFR) in swine. This method uses a specific correction formula applied to a one-compartment model of pharmacokinetic analysis. The procedure includes the following steps: (i) administering 6.47 g iohexol through an intravenous catheter placed at the marginal auricular vein; (ii) collecting six blood samples at 120, 180, 240, 300, 360, and 420 min after the iohexol injection; (iii) determining plasma iohexol concentrations by HPLC–UV; (iv) calculating the best fitting curve for these concentrations by a slope–intercept method; (v) calculating iohexol plasma clearance as the ratio of dose over area under the curve; and (vi) correcting the obtained value by a formula.

Our method was performed in swine with normal GFR, paving the way for future studies in animals with reduced GFR. However, the applicability of the method should not depend on the level of GFR. The mathematical approach will be the same for animals with normal, supranormal, or reduced GFR, as it is in humans [15,16]. Such a hypothesis is supported by the previous studies of Bröchner–Mortensen [24] who developed a formula in Caucasian patients that has subsequently been applied for all levels of renal function in many studies.

This new proposed protocol has major advantages: it is performed in conscious animals with no movement restrictions, it uses a single dose of the marker, it requires a reduced number of blood samples (*n* = 6), and it avoids urine collection. This represents a significant improvement in animal welfare conditions and handling necessities in experimental trials that require the evaluation of GFR. Importantly, our method eliminates the influence of sedation or anesthesia on GFR [25,26].

For the selection of new methods in research with animal models, sampling techniques must be reproducible and simple. Our method provides a simple and reliable approach for GFR measurement in swine, using only blood sample extractions. Urine sampling can be used in swine but implies catheterization of the urinary tract, which is technically difficult and requires the use of anesthetics [27,28,29].

The clearance of inulin is considered the gold standard method in the measurement of GFR. However, this method is neither simple nor practical, particularly in large animals. On the other hand, plasma clearance of iohexol has shown good correlation with the clearance of inulin [15,16]. In our method, urine collection is not necessary. This is a major simplification since the collection of urine is complicated in these animals. In swine, Frenbby et al. [21] reported a difference of 4.0 mL/min per 10 kg weight between renal and plasma clearances of iohexol. In this study, the last sample for calculating GFR was collected at 270 min, which may have influenced the results. The number and the timing for the last sample in multiple-sample approaches are fundamental to achieving acceptable precision and accuracy. The later the last sample is collected, the better is the concordance between renal and plasma clearances of iohexol [16]. In our study, the last sample was taken at 420 min, which may have led to a better agreement between our method and the urinary clearance of iohexol. In any case, urine collection would make the whole procedure much more complex and difficult to perform.

The plasma clearance of iohexol was originally described in 1984 in humans [30] and since then it has been frequently used in clinical research. Iohexol is a stable molecule, which is freely filtrated through the glomeruli; it is not metabolized by tubular cells and is completely eliminated in the urine [31]. Moreover, the procedure is very safe with few and minor side effects having been reported. Plasma clearance of iohexol is based on the disappearance curve of the marker, which typically fits a two-compartment model with an initial rapid reduction (*distribution phase*) followed by a slow and linear decline in plasma concentration (*elimination phase*). One-compartment models are focused only on the elimination phase, which has the advantages of limiting the number of samples for estimating GFR and improving animal welfare, handling necessities, and cost efficiency. However, this approach does not take into account the early distribution phase, which is a source of errors, making the use of a corrective formula necessary [24]. A previous study in swine [21] used a formula developed in humans, the Bröchner–Mortensen equation, to correct for the clearance derived from the one-compartment model [24]. However, this adjustment did not improve the performance of the simplified method. The formula of Bröchner–Mortensen was developed in humans in whom the values of GFR ranged from 0 to 120 mL/min [24]. In our study, the GFR values of the swine were two times higher or even more and this may be the reason why the Bröchner–Mortensen formula is not accurate in swine. In our study, we firstly evaluated a two-compartment model, which was considered as the *reference method*. The clearance obtained using the values of the elimination phase led to a 20–30% overestimation of the true GFR measured by the *reference method.* Then, we developed a correction formula to adjust the clearance of the elimination phase to the reference method. This *simplified method* importantly reduced the error to less than 10%.

Finally, we tested the agreement between the values of GFR obtained by the simplified method and the two-compartment model. The results of the Bland–Altman test, as well as the TDI and CCC, showed good agreement, which allows the use of this method in swine to evaluate GFR.

## 4. Material and Methods

### 4.1. Ethics Statement

The study was performed according to the Spanish Policy for Animal Protection RD1201/05, which meets the European Union Directive 86/609 about the protection of animals used in research. The experiment was specifically assessed and approved (report CEEA 2012/012, 28/01/2012) by the Instituto Nacional de Investigación y Tecnología Agraria y Alimentaria (INIA) Committee of Ethics in Animal Research, which is the named Institutional Animal Care and Use Committee (IACUC) for the INIA. The sows were housed at the animal facilities of the INIA, which meet local, national, and European requirements for Scientific Procedure Establishments.

### 4.2. Experimental Design

We used two groups of animals (test and validation) involving a total of 16 female adult Iberian swine (8 to 10 years old); each group was formed by eight animals. The animals were conscious throughout the experiment, were restrained only for sampling for 3 to 5 min, and were free to move throughout the experiment. No sedation or anesthesia was used for the sampling procedure.

At 8:00 a.m., after 6–8 h of fasting, a single dose of 10 mL Omnipaque 300 (GE Healthcare, Madrid, Spain) containing 6.47 g iohexol was injected for 2 min through the marginal auricular vein of one ear. We selected a single dose of 10 mL since it is the same dose we use in humans [15,16]. We prefer to avoid an infusion of the marker since this procedure would need the use of sedation or anesthesia. After injection, blood samples (3 mL) were taken at 15, 30, 45, 60, 90, 120, 180, 240, 300, 360, and 420 min and collected in EDTA-treated tubes. This protocol was based on Frennby et al. [21] with some modifications: samples were reduced from 16 extractions to 11 for the two-compartment model, and to six for the one-compartment model (see below). Blood samples were taken from the orbital sinus. Phlebotomy can be difficult in swine since there are few viable sites from which to draw blood; surface veins are small and the use of deep veins such as the cava and jugular is technically complex, which increases discomfort and the risk for bleeding, especially in big animals. Blood collection from the orbital sinus is an established technique in veterinary practice described in 1969 and is minimally invasive and simple [32,33]. Also, animals exhibit little discomfort and return to their activities after the procedure is completed. 

A blank (iohexol-free) blood sample was collected at time zero before the marker was administered. Blood samples were immediately centrifuged at 2000× *g* for 15 min and plasma was stored at −80 °C in the biobank.

### 4.3. Iohexol Measurements

Iohexol plasma concentrations were measured by HPLC–UV as previously reported [34]. Briefly, 200 μL of plasma was added to 50 μL of the internal standard (IS) 1,3-dimethyluric acid (DMU) (500 mg/mL) and deproteinized with 750 μL of 5% perchloric acid. Samples were vortexed and centrifuged for 5 min at 12,500 rpm. A 5 μL aliquot of supernatant was chromatographed by a C18 reversed-phase column (5 mm, 150 × 4.6 mm, Advanced Chromatography Technologies Ltd., Aberdeen, UK) using an HPLC system (Agilent Series 1260 Infinity, Santa Clara, CA, USA) equipped with a diode array detector set at 254 nm. Iohexol isomers were eluted by a mixture of deionized water/acetonitrile (96:4 by volume, adjusted to pH 2.5 with phosphoric acid) pumped at a flow rate of 1.0 mL/min. The calculations of the concentrations of iohexol were performed using the height of the peak of the second isomer of iohexol compared with the IS peak (peak height ratio).

### 4.4. Calibration and Quality Control Standards

Internal calibration curves of iohexol were prepared for each set of samples. A working solution of iohexol (647 µg/mL) was prepared in deionized water and used for the calibration curve and quality control samples. A total of five concentrations of iohexol (32.35, 64.7, 97.05, 129.4, and 161.75 µg/mL in plasma) were used as calibrators. Two quality control standards (QCs) prepared in-house, containing iohexol at low (64.7 µg/mL) and high (129.4 µg/mL) concentrations, were also prepared and used for validation tests. Calibrators, quality control samples, and reference standard solutions were stored at −20 °C until use.

### 4.5. Pharmacokinetic Analyses: One- and Two-Compartment Models

a. Two-compartment model (CL2): in the test group the concentrations of iohexol at 15, 30, 45, 60, 90, 120, 180, 240, 300, 360, and 420 min were fitted by nonlinear regression analysis to calculate the area under the curve (AUC). The iohexol plasma clearance was calculated as the ratio between the dose of iohexol and the AUC (dose/AUC). 

b. One-compartment model (CL1): in the test group only the elimination phase, which starts at 120 min after injection of the marker, was considered. Then, the concentrations of iohexol at 120, 180, 240, 300, 360, and 420 min were fitted by a slope–intercept method to determine the AUC. The slope–intercept method considers data only of the slow exponential and the fit is done by taking the natural logarithm of the plasma concentrations (Pi). The linear regression of ln(Pi) against the time (ti) was performed to determine the slope (–k) and the intercept (ln(P0)). The AUC of the single exponential is given by AUC = (P0)/k. The iohexol plasma clearance was determined as the ratio dose/AUC.

### 4.6. Developing a Correction Formula to Simplify the Method

GFR calculated by CL1 overestimated true GFR assessed by CL2 (Table 1). The one-compartment model (CL1) underestimated the AUC because it did not consider the initial distribution phase of iohexol. Thus, a formula was needed to recalculate the true clearance. Based on a previous publication [21], we tested the Bröchner–Mortensen equation to adjust the values of CL1. Different equations were developed to recalculate CL1 using linear and nonlinear regression models. The best equation was selected based on the highest *R*^2^. This equation was considered as the simplified method (SM) to measure GFR using the plasma clearance of iohexol. In the validation group, we calculated CL2 and CL1 and applied the SM as described above.

### 4.7. Reproducibility Study

The reproducibility of the plasma clearance of iohexol was determined in an extra group of 12 animals (2 to 3 years old) in which the method was performed two times, separated by seven days. We calculated the absolute difference of the method using the mean absolute percentage error.

### 4.8. Sensitivity Analysis

To evaluate the validity of the initial time point of the elimination phase, GFR was calculated considering the starting point at 120 min and at 180 min.

### 4.9. Pharmacokinetic Analysis

Results were expressed as mean ± SD. The fit between CL1 and CL2 was evaluated with several regression models: linear, logarithmic, inverse, quadratic, cubic, compound, power, S-curve, exponential, and logistic. All data were fitted by a nonlinear regression iterative program. The best equation was selected based on the higher R square and the lower differences between CL2 and CL1. The formula was applied to CL1 and this was considered the simplified method (SM) for the iohexol plasma clearance. Calculations and graphical representation were performed with SPSS Statistics for Windows, version 17.0 (SPSS Inc., Chicago, IL, USA).

### 4.10. Statistical Analysis: Tests of Agreement

The agreement between CL2 and SM was assessed by the limits of agreement described by Bland and Altman [35] and the total deviation index (TDI), concordance correlation coefficient (CCC), and coverage probability (CP) as proposed by Lin et al. [36]. The limits of agreement are a simple graphic tool which describes the limits that include the majority of the differences between two measurements. The narrower these limits are, the better the agreement. CCC combines elements of accuracy and precision. Its scores range from 0 to 1 and a value > 0.90 reflects optimal concordance between measurements. TDI is a measure that captures a large proportion of data within a boundary for allowed differences between two measurements [36]. CP ranges from 0 to 1; it is a statistic that estimates whether a given TDI is less than a pre-specified percentage [37]. The ideal situation is to have a TDI <10%, meaning that 90% of the estimations fall within an error of ±10% from the gold standard. Finally, these statistics provide confidence intervals that allow generalization of the results. 

For the Bland and Altman test, we used the MedCalc statistical software, version 15.8 (MedCalc Software bvba, Ostend, Belgium). For the agreement analyses, we used the statistical package AGP (Agreement Program) version 1.0 (IGEKO, SP) available at: http://lfr.ecihucan.es/apps/agreement_installer/Agreement_Installer.exe. The AGP is based on the R code originally developed by Lawrence Lin and YuYue [37]. The AGP was developed to simplify the use of the tool given in the R agreement package (R Core Team (2017). R: A language and environment for statistical computing. R Foundation for Statistical Computing, Vienna, Austria. URL https://www.R-project.org/.5. 

## 5. Conclusions

In conclusion, we have developed a *simplified method* to measure renal function in swine. This method is simple, reproducible and reliable, accurate and precise, requires a reduced number of blood samples, and improves animal management and welfare. Moreover, this new method facilitates sequential measurements of renal function, which allows the assessment of changes in GFR over time. Finally, the proposed protocol is similar to the one used in clinical research in humans, which could facilitate translational studies.

## Figures and Tables

**Table 1:** Iohexol plasma clearance in the one- (CL1) and two-compartment (CL2) models in the test and validation groups. SM = simplified method. BM = Bröchner–Mortensen equation.* mL/min. ** kg.

**Test Group**							
**CASE**	Weight **	CL2 *	CL1 *	SM *	**SM–CL2 (%)**	BM *	**BM–CL2 (%)**
1	113	119.6	150.4	114.6	−4.2	121.5	1.6
2	122	172.6	236.3	194.3	12.5	166.1	−3.8
3	138	225.8	301.6	248.7	10.1	188.1	−16.7
4	132	233.8	255.4	210.8	−9.9	173.6	−25.8
5	101	175.8	216.4	176.7	0.5	157.4	−10.5
6	210	341.8	433.1	341.4	−0.1	200.6	−41.3
7	209	300.5	345.6	282.1	−6.1	196.9	−34.5
8	212	392.0	527.4	394.4	0.6	183.7	−53.1
mean ± SD	155 ± 47	245 ± 93	308 ± 123	245 ± 91		190 ± 26	
**Validation Group**							
	Weight **	CL2 *	CL1 *	SM *	**SM–CL2**	BM *	**BM–CL2**
9	106	173.7	213.7	174.2	0.3	156.1	−10.2
10	182	246.8	275.9	227.9	−7.7	180.7	−26.8
11	116	112.1	128.0	92.2	−17.8	106.9	−4.7
12	159	289.9	394.6	316.6	9.2	201.3	−30.6
13	156	202.8	253.5	209.1	3.1	172.9	−14.8
14	176	289.6	331.2	271.4	−6.3	194.5	−32.8
15	115	202.8	257.7	212.7	4.9	174.4	−14.0
16	188	313.9	360.9	293.2	−6.6	198.9	−36.6
mean ± SD	150 ± 33	229 ± 69	277 ± 85	225 ± 71		181 ± 31	

## Supplementary Materials

Supplementary materials can be found at www.mdpi.com/1422-0067/19/1/232/s1.

## References

[1] Houdebine L.M., Hedrich H. (2004). The Mouse as an Animal Model for Human Diseases. The Laboratory Mouse.

[2] Muhammad S. (2014). Nephrotoxic nephritis and glomerulonephritis: Animal model versus human disease. Br. J. Biomed. Sci..

[3] Betz B., Conway B.R. (2014). Recent advances in animal models of diabetic nephropathy. Nephron Exp. Nephrol..

[4] Herrmann S.M., Sethi S., Fervenza F.C. (2012). Membranous nephropathy: The start of a paradigm shift. Curr. Opin. Nephrol. Hypertens..

[5] Taylor C.M., Williams J.M., Lote C.J., Howie A.J., Thewles A., Wood J.A., Milford D.V., Raafat F., Chant I., Rose P.E. (1999). A laboratory model of toxin-induced hemolytic uremic syndrome. Kidney Int..

[6] Yokota S.D., Benyajati S., Dantzler W.H. (1985). Comparative aspects of glomerular filtration in vertebrates. Ren. Physiol..

[7] Davies B., Morris T. (1993). Physiological parameters in laboratory animals and humans. Pharm. Res..

[8] Sachs D.H. (1994). The pig as a potential xenograft donor. Vet. Immunol. Immunopathol..

[9] Tumbleson M.E., Schook L.B., Tumbleson M.E., Schook L.B. (1996). Advances in Swine in Biomedical Research.

[10] Cibulskyte D., Pedersen M., Hjelm-Poulsen J., Hansen H.E., Madsen M., Mortensen J. (2006). The pharmacokinetics and acute renal effects of oral microemulsion ciclosporin A in normal pigs. Int. Immunopharmacol..

[11] Lodrup A.B., Karstoft K., Dissing T.H., Nyengaard J.R., Pedersen M. (2008). The association between renal function and structural parameters: A pig study. BMC Nephrol..

[12] Kidney Disease Outcome Quality Initiative (2002). Clinical practice guidelines for chronic kidney disease: Evaluation, classification and stratification. Am. J. Kidney Dis..

[13] GBD 2013 Mortality and Causes of Death Collaborators (2015). Global, regional, and national age-sex specific all-cause and cause-specific mortality for 240 causes of death, 1990–2013: A systematic analysis for the Global Burden of Disease Study 2013. Lancet.

[14] Levey A.S., Inker L.A., Coresh J. (2014). GFR estimation: From physiology to public health. Am. J. Kidney Dis..

[15] Delanaye P., Melsom T., Ebert N., Bäck S.E., Mariat C., Cavalier E., Björk J., Christensson A., Nyman U., Porrini E. (2016). Iohexol plasma clearance for measuring glomerular filtration rate in clinical practice and research: A review. Part 2: Why to measure glomerular filtration rate with iohexol?. Clin. Kidney J..

[16] Delanaye P., Ebert N., Melsom T., Gaspari F., Mariat C., Cavalier E., Björk J., Christensson A., Nyman U., Porrini E. (2016). Iohexol plasma clearance for measuring glomerular filtration rate in clinical practice and research: A review. Part 1: How to measure glomerular filtration rate with iohexol?. Clin. Kidney J..

[17] Odlind B., Hällgren R., Sohtell M., Lindström B. (1985). Is 125I iothalamate an ideal marker for glomerular filtration?. Kidney Int..

[18] Zurth C. (1984). Mechanism of renal excretion of various X-ray contrast materials in rabbits. Investig. Radiol..

[19] Nilsson-Ehle P., Grubb A. (1994). New markers for the determination of GFR: Iohexol clearance and cystatin C serum concentration. Kidney Int. Suppl..

[20] Donadio C., Tramonti G., Giordani R., Lucchetti A., Calderazzi A., Bassani L., Bianchi C. (1990). Effects on renal hemodynamics and tubular function of the contrast medium iohexol in renal patients. Ren. Fail..

[21] Frennby B., Sterner G., Almén T., Chai C.M., Jönsson B.A., Månsson S. (1997). Clearance of iohexol, 51Cr-EDTA and endogenous creatinine for determination of glomerular filtration rate in pigs with reduced renal function: A comparison between different clearance techniques. Scand. J. Clin. Lab. Investig..

[22] Lundqvist S., Hietala S.O., Karp K. (1993). Experimental studies comparing iohexol and 51Cr-EDTA for glomerular filtration rate measurements. Acta Radiol..

[23] Russell W.M. (1969). The development of the Anim Care. Lab. Anim. Care.

[24] Bröchner-Mortensen J. (1972). A simple method for the determination of glomerular filtration rate. Scand. J. Clin. Lab. Investig..

[25] Colson P., Saussine M., Séguin J.R., Cuchet D., Chaptal P.A., Roquefeuil B. (1992). Hemodynamic effects of anesthesia in patients chronically treated with angiotensin converting enzyme inhibitors. Anesth. Analg..

[26] Fusellier M., Desfontis J.C., Madec S., Gautier F., Debailleul M., Gogny M. (2007). Influence of three anesthetic protocols on glomerular filtration rate in dogs. Am. J. Vet. Res..

[27] Finco D.R., Braselton W.E., Cooper T.A. (2001). Relationship between plasma iohexol clearance and urinary exogenous creatinine clearance in dogs. J. Vet. Intern. Med..

[28] Gaspari F., Perico N., Matalone M., Signorini O., Azzollini N., Mister M., Remuzzi G. (1998). Precision of plasma clearance of iohexol for estimation of GFR in patients with renal disease. J. Am. Soc. Nephrol..

[29] Miyagawa Y., Takemura N., Hirose H. (2010). Evaluation of a single sampling method for Estimation of plasma iohexol clearance in dogs and cats with various kidney functions. J. Vet. Med. Sci..

[30] Krutzén E., Bäck S.E., Nilsson-Ehle I., Nilsson-Ehle P. (1984). Plasma clearance of a new contrast agent, iohexol: A method for the assessment of glomerular filtration rate. J. Lab. Clin. Med..

[31] Rocco M.V., Buckalew V.M., Moore L.C., Shihabi Z.K. (1996). Measurement of glomerular filtration rate using nonradioactive Iohexol: Comparison of two one-compartment models. Am. J. Nephrol..

[32] Dove C.R., Alworth L.C. (2015). Blood collection from the orbital sinus of swine. Lab. Anim..

[33] Huhn R.G., Osweiler G.D., Switzer W.P. (1969). Application of the orbital sinus bleeding technique to swine. Lab. Anim. Care.

[34] Luis-Lima S., Gaspari F., Porrini E., García-González M., Batista N., Bosa-Ojeda F., Oramas J., Carrara F., González-Posada J.M., Marrero D. (2014). Measurement of glomerular filtration rate: Internal and external validations of the iohexol plasma clearance technique by HPLC. Clin. Chim. Acta.

[35] Bland J.M., Altman D.G. (1986). Statistical methods for assessing agreement between two methods of clinical measurement. Lancet.

[36] Lin L., Hedayat A., Wu W. (2012). Statistical Tools for Measuring Agreement.

[37] Lin L., Hedayat A., Sinha B., Yang M. (2002). Statistical methods in assessing agreement: Models, issues, and tools. J. Am. Stat. Assoc..

