# Supplementary material for: A Simple Method to Measure Renal Function in Swine by the Plasma Clearance of Iohexol
Source: Int J Mol Sci. 2018 Jan 12;19(1):232. doi: 10.3390/ijms19010232 (PMC5796180; doi:10.3390/ijms19010232)
Supplement: Supplementary file 1 [file ijms-19-00232-s001.pdf]

Supporting information

S1 Table. Regression models. 10 linear and non-linear regression models were developed

Equation	Resume of the models					Regression coefficients			
	R square	F-Snedecor	DF 1	DF 2	*P*-Value	Intercept	Beta 1	Beta 2	Beta 3
Linear	0.96	141.7	1	6	<0.001	17.885	0.737		
Logaritmic	0.95	127.6	1	6	<0.001	-1035.718	226.259		
Inverse	0.87	39.9	1	6	0.001	459.672	−57505.551		
Quadratic	0.97	77.1	2	5	<0.001	−47.909	1.176	-6.3968×10^{-4}	
Cubic	0.97	43.3	3	4	0.002	23.880	0.407	0.002	-2.493×10^{-6}
Compound	0.90	54.8	1	6	<0.001	89.741	1.003		
Power	0.96	148.9	1	6	<0.001	0.960	0.967		
S-curve	0.94	98.3	1	6	<0.001	6.388	−255.2		
Exponential	0.90	54.8	1	6	<0.001	89.741	0.003		
Logistic	0.90	54.8	1	6	<0.001	0.011	0.997		

S2 Table. Reproducibility study. Iohexol plasma clearance for the Simplified method (SM) in two occasions in two occasions on two weeks apart in a group of 12 adult iberian pigs. The precision (time-to-time variability) was evaluated as mean absolute percentage error (MAPE) of GFR (ml/min) for each case.

PIG ID	GFR REPLICA 1	GFR REPLICA 2	MAPE
1	131.3	126.4	3.7
2	122.9	128.5	4.5
3	175.5	151.1	13.9
4	146.5	121.8	16.8
5	221.7	185.5	16.4
6	142.4	146.6	2.9
7	154.2	167.1	8.4
8	188.1	191.1	1.6
9	136.5	155.3	13.8
10	202.4	217.7	7.6
11	164.5	184.0	11.8
12	245.6	219.8	10.5
**Mean**	**169.3**	**166.3**	**9.3**
